# Intellectual property management at the National Animal Science Research Institute in India: A case study

**DOI:** 10.14202/vetworld.2019.1070-1077

**Published:** 2019-07-20

**Authors:** C. Chithra Chandrasekharan, R. S. Jiji

**Affiliations:** Department of Veterinary and Animal Husbandry Extension, College of Veterinary and Animal Sciences, Thrissur, Kerala, India

**Keywords:** Indian Council of Agricultural Research, intellectual property management system, intellectual property rights, National Institute of Veterinary Epidemiology and Disease Informatics, strengths, weaknesses, opportunities, and threats analysis

## Abstract

**Aim:**

The National Institute of Veterinary Epidemiology and Disease Informatics is an animal science research institute under the aegis of the Indian Council of Agricultural Research. The intellectual property management system (IPMS) of the institute oversees technology creation, protection, and transfer/commercialization. This study reviews the effectiveness of the IPMS using traditional strengths, weaknesses, opportunities, and threats (SWOT) evaluation.

**Materials and Methods:**

A comprehensive repository was developed to compile the SWOT pertaining to the IPMS based on relevant document reviews and the inputs of experts and stakeholders. The repository was shared among scientists of the institute for rating. The rating process revealed the top ten key SWOT associated with the structure and operation of the IPMS. The weighted SWOT matrix technique was used to identify the best strategies to improve and develop the IPMS further. This included strategies derived from the best combinations of key strengths and opportunities (S-O strategies), key weaknesses and opportunities (W-O strategies), key strengths and threats (S-T strategies), and key weaknesses and threats (W-T strategies).

**Results:**

The top-ranked strengths included “possession of patented technology” and “state-of-the-art biosafety laboratory facilities,” while “lack of in-house faculty with legal expertise in intellectual property rights (IPR)” and “lack of technology incubation facilities” were the key weaknesses. The key opportunities included “external funding for research projects” and “market demand for onsite diagnostic tools.” The major threats were “lack of market for veterinary diagnostics” and “broad-based patents on research tools and technologies.”

**Conclusion:**

The strengths of the system, such as a state-of-the-art biosafety laboratory and technology-marketing collaboration with Agrinnovate India Ltd., could be employed effectively to gain from the opportunities tendered by the market demand for on-site disease diagnostic tools (S-O strategies). The limitation arising from a dearth of technical staff could be overcome by technological backstopping through international linkages in the area of disease monitoring and surveillance. Funding from externally supported projects could also be utilized for recruitment of personnel (W-O strategies). Limitations arising from the combination of inadequate in-house IPR expertise and the threat arising from broad-based patents on research tools warrant vigilance (W-T strategies).

## Introduction

Intellectual property rights (IPR) have widely been recognized as a strategic tool for technology and innovation management in academic and research operations. The Trade Related Aspects of Intellectual Property Rights Agreement (TRIPS) advocates member countries to bring in minimum standards for intellectual property protection in all technology fields including those of the agricultural and livestock sectors [1]. In India’s developing economic landscape, a world-class IPR management structure is necessary to protect and encourage technology research, innovation, and commercialization. In public sector research organizations and universities in India, there have been ample opportunities for translation of IPR-enabled technologies into commercial success, at par with competent private sector entities. The National IPR Policy of India [2] envisages strengthening the academia/industry interface to promote functional collaboration of ideas and research along with technology commercialization and entrepreneurship development. The science, technology and innovation policy [3] also embraces enhanced participation of the private sector in research and development and channeling research outputs into commercial and societal applications. Public-Private Partnership (PPP) strategies enable public sector research organizations to engage in collaborative research besides joint validation, refinement, up-scaling, and technology marketing. The monetary benefits, including licensing fees and royalties that accrue from commercialized technologies, could effectively be utilized to incentivize innovators and researchers as well as strengthen the research infrastructure. This approach envisages instilling an innovative research environment for academia and fostering investment in research and development activities. The Indian Council of Agricultural Research (ICAR), the nodal organization of the National Agricultural Research System (NARS), has established a customized policy framework and institutional mechanism for the protection and transfer or commercialization of its intellectual property resources [4]. The organization comprises a three-tier structure with Institute Technology Management Units (ITMUs) at the individual institute level as the base layer, five Zonal Technology Management Centres (ZTMCs) in selected institutes at the zonal level as the intermediate layer, and the Agro Technology Management Centre at the central level as the apex layer. The intellectual property and technology management unit (IP and TM unit) at the ICAR headquarters oversees the functioning of these bodies. The ZTMCs of different zones facilitate and coordinate the functioning of the ITMUs in the respective zones. The south zone ZTMC situated in the Central Institute of Fisheries Technology coordinates the activities of the ITMUs of twenty-two ICAR research institutes including that of the National Institute of Veterinary Epidemiology and Disease Informatics (NIVEDI). The functional decentralization strategy, as devised by the guidelines, has entrusted the IPR portfolios of the individual institutes with ample powers and internal capabilities.

The IPR portfolio of ICAR can be a model for other constituent units of NARS, including state veterinary universities and other research entities, for the strategic development and management of technologies aimed at social, environmental, and economic benefits to the industry. An in-depth study of this model is essential to address the specific features and requisites of the livestock sector. The present study assumes significance in this context.

This study investigates the strengths, weaknesses, opportunities, and threats (SWOT) pertaining to the intellectual property management system (IPMS) of NIVEDI, a south zone animal sciences research institute of ICAR. The mission of this institute is epidemiological surveillance and monitoring of nationally important livestock and poultry diseases through effective networking and capacity building.

## Materials and Methods

### Ethical approval and informed consents

The approval to conduct this study was obtained through the following permissions: Proceedings of the Kerala Veterinary and Animal Sciences University with Approval No. KVASU/DAR/Acad A (l)/l1795/2014 dated May 30, 2014; research grants approval of the Dean, College of Veterinary and Animal Sciences, Mannuthy through Order No. Acad (3) 6116/2014 and permission letter from the Director, NIVEDI, dated May 25, 2015.

### Procedures used

The SWOT pertaining to the IPMS of NIVEDI was analyzed based on the procedures and methods devised by Weihrich [5], Collado *et al*. [6], and Lu [7]. The SWOT analysis was carried out in four successive phases as follows.

### Defining the institutional IPMS

The IPMS was operationally defined as the system that performed the functions of intellectual property creation, protection, and transfer/commercialization.

The internal and external environmental factors influencing the system’s functioning were broadly categorized. The internal factors were recognized as those elements that could be controlled and modified to manage the institutional IP more efficiently. The internal factors comprised both strengths and weaknesses. “Strengths” were recognized as factors that could be utilized for efficient management of intellectual property. “Weaknesses” exposed aspects that could be eliminated or minimized for efficient functioning of the system.

The external factors included socioeconomic, political, and environmental elements that were generally beyond the control of the IPMS. Within the context of the SWOT analysis, “Opportunities” referred to the external factors that improved the performance of the IPMS, whereas “Threats” were elements that impeded performance.

### Identification and categorization of the SWOT factors

Based on the inputs received from a review of documentary evidences, discussions with experts, and a review of relevant studies, a comprehensive repository of the internal (strengths and weaknesses) and external (opportunities and threats) factors was created for the IPMS, grouping the factors relative to their respective domains. The factors pertaining to strengths and weaknesses were classified under four-factor domains: Technology, infrastructure, human resources, and technology transfer/marketing strategies. For both opportunities and threats, the factor domains defined were socioeconomic, policy, market, and outside organizations. The repository was further refined to reflect views and recommendations resulting from focus group discussions and personal/telephonic interviews with scientists and other stakeholders, who were selected based on the suggestions of the ITMU personnel.

In focus group discussions with the scientists of the institute, the researcher presented the SWOT repository and invited discussion and suggestions. Modifications were made to the repository to reflect the ideas and suggestions of the scientists. In addition, the inputs received from selected stakeholders, including industry personnel, scientists from sister organizations, professional associations, and representatives from policymaking bodies such as the National Academy of Agricultural Research Management, were considered for fine-tuning the SWOT repository.

Following all revisions, the final SWOT repository comprised 41 strengths (S), 22 weaknesses (W), 41 opportunities (O), and 23 threats (T).

### Validation of the SWOT items

The substantial number of factors that comprised the SWOT repository was condensed into more focused ones through validation. While the SWOT repository was developed with inputs from multiple stakeholder groups, validation of the SWOT factors was performed following a rating procedure, involving the institute’s scientists who were directly involved in IP creation, protection, and transfer/commercialization.

The respondents were asked to rate the SWOT factors on a four-point Likert scale, namely, strongly agree, agree, somewhat agree, and disagree with scores of 4, 3, 2, and 1, respectively. The summation of scores assigned by all the respondents for a particular SWOT factor indicated the factor’s score. The mean score of the factor was calculated by applying the formula:


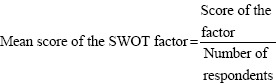


In the score-based ranking hierarchy, the top ten rankings in each factor category were identified as the most influential or key factors.

### Identification and prioritization of strategies for effective functioning and development of IPMS

In this phase, a weighted/quantitative SWOT matrix technique was employed to further enrich the output of the SWOT analysis.

**Figure-1:**
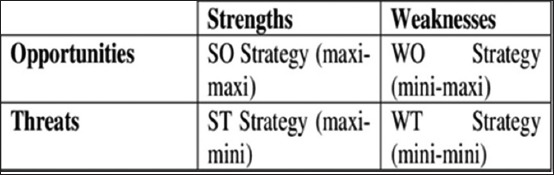
Strategic options based on strengths, weaknesses, opportunities, and threats matrix.

The TOWS matrix, also referred to as the SWOT matrix, was originally proposed by Weihrich [5] for matching the external opportunities and threats of an organization with its internal strengths and weaknesses. The interaction matrix could further provide alternative strategies for decision-making/problem-solving in an organization. Figure-1 illustrates the strategies proposed by Weihrich. The strategies were based on an interaction of the internal and external factors. Accordingly, the four strategic options would be maxi-maxi (utilize strengths to take advantage of opportunities), maxi-mini (use strengths to minimize or reduce the impact of threats), mini-maxi (overcome weaknesses using opportunities), and mini-mini (minimize weaknesses and reduce the impact of threats). As opined by Weihrich [5], any organization, including government organizations, to be effective, should use such rational approaches for anticipating, responding to, and even altering the future environment.

**Figure-2:**
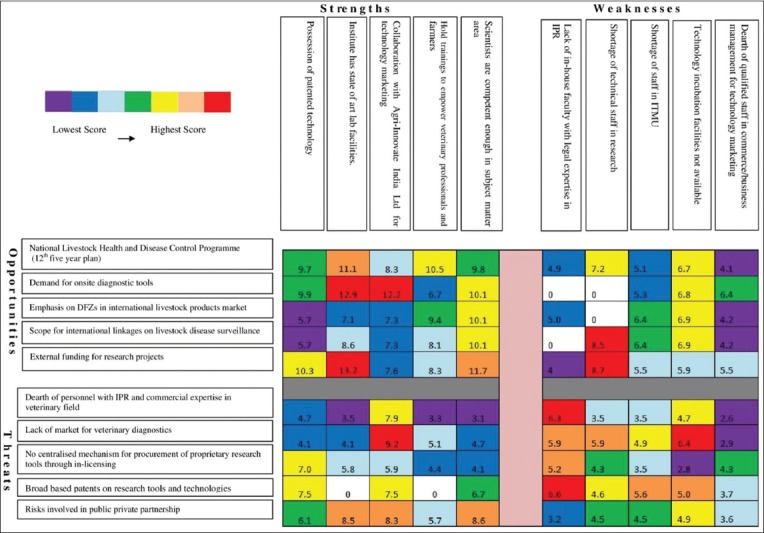
Weighted strengths, weaknesses, opportunities, and threats matrix showing strategies (S-O, W-O, S-T, and W-T) for further development of intellectual property management system of National Institute of Veterinary Epidemiology and Disease Informatics.

The present study, besides identifying the most influential driving and inhibiting factors, carried out an in-depth analysis of the relationship between these factors to derive certain strategic decisions for the refinement and further development of the IPMS. Accordingly, based on the SWOT analysis, quantitative interaction matrices of key factors were developed (Figure-2).

The matrix (Figure-2) entailed matching the five key internal factors with top scores under the strength and weakness categories with the corresponding external factors under the opportunity and threat categories. The coefficient (r) developed by Lu [7] was used to indicate the degree of matching/relationship between any two SWOT factors, wherein r=1 meant a perfect match, r=0 showed a non-existent relationship, and 0<r<1 denoted different degrees of relationship ranging from non-relationship to a perfect match. The coefficients were assigned based on consensus among the IPMS authorities. Subsequently, for the SWOT factor pairs that were matched, composite scores were computed using the formula: Composite score = Product of the score values of internal and external factors matched × coefficient (r).

The degree of importance of the consequent strategies was ascertained based on the composite scores. The scores were fed into the corresponding cells of the matrix. Further, the matrix cells were shaded with color gradations as in the “VIBGYOR” spectrum ranging from red to violet in the decreasing order of importance.

## Results and Discussion

The findings of the study are presented in Tables-1 and 2 and matrix forms (Figure-2). Data in Table-1 reveal the ten top ranking strengths and weaknesses of the IPMS in NIVEDI, based on rating by the respondents, whereas Table-2 depicts the ten top ranking opportunities and threats.

**Table 1:** Perceived importance of internal factors affecting the intellectual property management system.

Sl. No	Strengths	Score	Rank	Weaknesses	Score	Rank
1.	Possess patented technology	3.89	I	Lack of in-house faculty with legal expertise in IPR	3.44	I
2.	State of the art biosafety laboratory facilities			Technology incubation facilities not available	3.11	II
3.	Hold training to empower veterinary professionals and farmers	3.67	II	Dearth of qualified staff in commerce business management for technology marketing	2.89	III
4.	Scientists are competent enough in the subject matter area	3.44	III	Shortage of staff in ITMU		
5.	Collaboration with Agri Innovate India Ltd. for technology marketing	3.33	IV	Shortage of technical staff in research		
6.	Systematic maintenance of digitized documents in ITMU	3.22	V	High input cost of diagnostics technologies	2.78	IV
7.	Tailor-made technologies for veterinary diagnostics industry developed			Technology commercialization at infant stage only		
8.	ITMU with facility for outsourcing attorney for patent filing	3.11	VI	Limited number of innovative technologies	2.67	V
9.	Multi-disciplinary teamwork through interdepartmental and inter-institutional research			Commercially viable technologies are scarce	2.56	VI
10.	Favorable attitude of scientists toward IPR enforcement			Inadequate infrastructure for market watch mechanism to monitor commercial prospects of technologies	2.33	VII

IPR=Intellectual property rights, ITMU=Institute Technology Management Unit

**Table 2:** Perceived importance of external factors affecting the intellectual property management system.

Sl. No	Opportunities	Score	Rank	Threats	Score	Rank
1.	External funding for research projects	3.78	I	Lack of market for veterinary diagnostics	3.44	I
2.	Demand for onsite diagnostic tools	3.67	II	Broad-based patents on research tools and technologies	3.22	II
3.	Emphasis on disease-free zones in the international livestock products market			Risks involved in Public private partnership	3.11	III
4.	Scope for international linkages on livestock disease surveillance			No centralized mechanism for procurement of proprietary research tools through in-licensing	3.0	IV
5.	National Livestock Health and Disease Control Programme (12^th^ 5-year plan)	3.56	III	Lack of personnel with legal and commercial expertise in veterinary field	3.0	
6.	Participation in the control and eradication of economically important livestock diseases	3.44	IV	Genetic variability of microorganism	2.89	V
7.	ICAR guidelines in place to facilitate IP management in the institute			Lack of thrust on IPR training to faculty		
8.	Patentability of innovative technologies in diagnostics			Risks of large investment in diagnostics industry		
9.	Scope for harnessing technological advancements in areas of satellite imaging, GIS, IT, etc., in disease monitoring and surveillance			High cost of securing and maintaining IPR	2.78	VI
10.	National Agricultural Innovation Project for setting up Business Planning and Development Units in ICAR institutes	3.33	V	Illegal animal trade		

GIS=Geographic information systems, IP=Intellectual property, ICAR=Indian Council of Agricultural Research

### Perceived strengths

An important example of strength was the institute-owned patented technology of a diagnostic kit that identifies brucellosis in small ruminants. The institute initiated the marketing of this technology utilizing a PPP in collaboration with Agrinnovate India Limited. The success of the technology and its commercialization suggests a representative model for using IPR enabled/patented IP as a valuable technology marketing tool. The institute also claims to have developed other technologies for the veterinary diagnostics industry.

Securing IPR protection such as patents to protect agricultural technologies developed by universities and research institutions has generally been recognized as a powerful tool that aids in the development and commercialization of research-driven innovation [8]. The innovation, knowledge, and technology transfer model has been described as the bridge between academia and the market by other researchers [9]. The newly established state-of-the-art biosafety laboratory, competent scientists with favorable attitudes toward IPR and the collaborative research culture were some other significant strengths perceived. The major strengths reported in the IP protection space included a well-established ITMU with systematic document management system and access to outsourced legal experts for patent filing.

### Perceived weaknesses

Many strategists assert that the IPR policies of universities and public/private sector institutions should prioritize the development of skills and processes to manage IPR and to leverage their value [10]. Among the major weaknesses perceived, the lack of in-house legal expertise in IPR was the most emphasized. The shortage of technical staff in research was also an issue of concern.

Despite its claim of developing an array of technologies for veterinary diagnostics, the institute faced many bottlenecks at the commercialization phase. Reportedly, technology commercialization was represented as being at an infant stage, mainly due to the lack of innovation and perceived commercial viability of technologies. The input cost of diagnostic technology development was reportedly high. In addition, the lack of qualified staff in commercial/business management and inadequate infrastructure for both market development and business incubation were reported as barriers. However, the very recent collaboration with Agrinnovate India Ltd. was viewed as a futuristic avenue for technology transfer and commercialization.

### Perceived opportunities

Access to research funding due to a significant number of funding agencies in public and private sectors was perceived as the most significant opportunity, as evidenced by the substantial number of externally aided projects under implementation at the institute.

The scientists seemed hopeful regarding the potential demand for on-site diagnostic tools like the ones devised by the institute. A policy document on options for livestock biotechnologies in developing countries refers to point-of-care diagnostic tests as significantly useful new tools for livestock farmers [11].

The factor relating to the strategic importance of disease-free zones in the international livestock products market was rated high by scientists. The knowledge and technologies generated in epidemiology and the diagnosis, monitoring, and surveillance of livestock diseases in India were instrumental in the formulation and implementation of the foot-and-mouth disease control program with the creation of disease-free zones [12]. The free trade regime insisting on national and international monitoring of diseases has opened avenues for NIVEDI to establish alliances with various stakeholders including veterinary institutions and colleges, diagnostic laboratories, and international organizations such as Food and Agricultural Organization, World Health Organization, and Office International des Epizooties or World Organization for Animal Health [13]. The scientists observed great opportunities in establishing linkages with eminent international players.

There was overwhelming appreciation among the scientists about the strengthening of the ambitious “National Livestock Health and Disease Control Programme” under the 12^th^ 5-year plan with added components for establishing disease diagnostic laboratories all over the country [14].

The scientists seemed aware of the opportunities offered by technology advancements in other niche areas (satellite imaging, geographic information systems, IT, etc.) in developing innovative disease monitoring and surveillance technologies and systems. The ICAR guidelines for IP management and the provision for Business Planning and Development units under the National Agricultural Innovation Project (now replaced by the agribusiness incubation centers under National Agricultural Innovation Fund Component II) were viewed as opportunities to develop synergies with IPMS.

The scientists seemed to understand the profound implications of the patentability of disease diagnostic kits according to patent laws [15].

### Perceived threats

Among the various threats perceived, the most challenging one was the poor market acceptance for veterinary diagnostics. A policy document on veterinary vaccines and diagnostics by National Academy of Agricultural Sciences [16] reported that though world-class vaccines and diagnostics had been developed against some important animal diseases by public sector R&D organizations, there were few manufacturers of these diagnostics in the country due to high input costs and lack of affordability for livestock owners.

Taylor and Cayford [17] reported that academic scientists often identified problems of access to vital technologies that impeded their agricultural research. The risks perceived by the scientific community pertaining to broad-based patents and the subsequent lack of freedom to operate are particularly relevant in relation to biotechnology research undertaken by R&D institutions like NIVEDI.

Despite the launch of some successful PPPs in product development and marketing, the scientists indicated apprehensions about the risks involved in PPPs. Spielman *et al*. [18] pointed out that PPPs carry some very unique risks, including those related to the coordination of collaborators with diverse interests, protection of research mandates and missions, integrity of firms and research centers, and exchange of proprietary knowledge. Perceived strategic negligence in IPR training, as one of the key issues, was a concern for the scientific community. The scientists also expressed concern over the complex process of patent filing, including the high cost involved.

### Strategies for the future development of NIVEDIs IPMS

#### S-O strategies

The strategies emerging from the unique blend of strengths and opportunities (Figure-2) describe how well the system’s strengths could be used to best advantage.

The research capability in niche areas, attributable to state-of-the-art infrastructure and expertise, would be instrumental in attracting research funds from various sources to create new research entities and to strengthen existing ones for both academic and economic sustainability [19].

The need and demand for cost-effective, onsite detection, pen side format veterinary diagnostics kits in the field have been reiterated in many reports on account of its simplicity and user-friendliness for farmers [16,20]. The institute’s enabling research infrastructure, together with marketing support from Agrinnovate India Ltd., could be leveraged to exploit market demand. In medicine, progress hinges on the successful translation of basic science discoveries into innovative diagnostics, medical devices, and therapeutics [21].

Further, provisions under the centrally-sponsored “National Livestock Health and Disease Control Programme” could be deployed for strengthening existing research entities.

#### W-O strategies

Limitations due to insufficient technical staff could be addressed by collaboration through international linkages in the fields of disease monitoring and diagnostics. The opportunities derived from externally aided projects could also be utilized for recruitment of personnel.

#### S-T strategies

The institute’s collaboration with Agrinnovate India Ltd., casually referred to as the “commercial arm of ICAR,” has reportedly boosted morale and enhanced confidence to engage in strategic ties with private sector industries. A relationship with an experienced, well-funded, private company could mitigate researchers’ concerns regarding the risks associated with PPPs. The innovation and technology transfer overlap has been observed as a dominant phenomenon in society by some researchers [22]. Mysore [8] opined that it is advantageous for beginners in technology transfer and commercialization to have a public sector mediator when licensing technologies to private entrepreneurs. However, factors such as the scientists’ perception of their own competence and an enabling and empowering facilitator, such as a private sector partner, could be instrumental in mitigating the uncertainties and fears surrounding PPPs.

#### W-T strategies

The constraint arising from the lack of veterinary personnel with any expertise in IPR, or with commercial expertise, both within and outside the system warrants attention. Intense training of scientists in patent research skills and freedom to operate analysis is a strategic imperative.

Similarly, the lack of an in-house institutional mechanism to promote research products through business incubation adds to the issue of access to markets for innovative products generated by the institute. In light of this, the system could either revamp the in-house infrastructure for technology marketing or actively pursue its strategic ties with Agrinnovate India Ltd.

## Conclusion

The findings of the study imply that capacity building of the scientific community in technology and IPR management deserves strategic emphasis to strengthen the IPR portfolio of the research institute. The marketability of technological products could be enhanced through strengthening in-house or outsourced market watch/intelligence interventions as well as institute-inspired start-ups. The IPR management capabilities would benefit from an in-house patent advisory capability for assessment of the patentability of innovations and freedom to operate in complex, innovative environments. The implementation of centralized procurement and in-licensing of proprietary research tools, as envisaged by the ICAR policy framework, would be the most practical strategy to address threats raised by broad-based patents, valid patents hidden in complex portfolios, and escalating research costs. Besides, creation of institutional platforms to facilitate policy discussions on the techno-legal and ethical issues surrounding IPR has become a strategic imperative in the backdrop of roadblocks to research, such as the “tragedy of anticommons” regarding patents blocks, broad-based patents, misuse of monopoly rights, and ethical considerations in bio patenting.

## Authors’ Contributions

CCC carried out the research work under the guidance of RSJ, who conceived and designed the work. CCC and RSJ have read and approved the final manuscript.
